# Supplementary material for: HIVprotI: an integrated web based platform for prediction and design of HIV proteins inhibitors
Source: J Cheminform. 2018 Mar 9;10:12. doi: 10.1186/s13321-018-0266-y (PMC5845081; doi:10.1186/s13321-018-0266-y)
Supplement: Supplementary file 2 — Additional file 2. Source code of HIVProtI web server. [file 13321_2018_266_MOESM2_ESM.zip › HIVprotI_Source-code/hivprotI/weka-3-6-6/doc/overview-frame.html]

Overview List


All Classes

Packages
  
weka.associations
  
weka.associations.gsp
  
weka.associations.tertius
  
weka.attributeSelection
  
weka.classifiers
  
weka.classifiers.bayes
  
weka.classifiers.bayes.blr
  
weka.classifiers.bayes.net
  
weka.classifiers.bayes.net.estimate
  
weka.classifiers.bayes.net.search
  
weka.classifiers.bayes.net.search.ci
  
weka.classifiers.bayes.net.search.fixed
  
weka.classifiers.bayes.net.search.global
  
weka.classifiers.bayes.net.search.local
  
weka.classifiers.evaluation
  
weka.classifiers.functions
  
weka.classifiers.functions.neural
  
weka.classifiers.functions.pace
  
weka.classifiers.functions.supportVector
  
weka.classifiers.lazy
  
weka.classifiers.lazy.kstar
  
weka.classifiers.meta
  
weka.classifiers.meta.nestedDichotomies
  
weka.classifiers.mi
  
weka.classifiers.mi.supportVector
  
weka.classifiers.misc
  
weka.classifiers.pmml.consumer
  
weka.classifiers.rules
  
weka.classifiers.rules.part
  
weka.classifiers.trees
  
weka.classifiers.trees.adtree
  
weka.classifiers.trees.ft
  
weka.classifiers.trees.j48
  
weka.classifiers.trees.lmt
  
weka.classifiers.trees.m5
  
weka.classifiers.xml
  
weka.clusterers
  
weka.clusterers.forOPTICSAndDBScan.Databases
  
weka.clusterers.forOPTICSAndDBScan.DataObjects
  
weka.clusterers.forOPTICSAndDBScan.OPTICS\_GUI
  
weka.clusterers.forOPTICSAndDBScan.Utils
  
weka.core
  
weka.core.converters
  
weka.core.logging
  
weka.core.mathematicalexpression
  
weka.core.matrix
  
weka.core.neighboursearch
  
weka.core.neighboursearch.balltrees
  
weka.core.neighboursearch.covertrees
  
weka.core.neighboursearch.kdtrees
  
weka.core.pmml
  
weka.core.stemmers
  
weka.core.tokenizers
  
weka.core.xml
  
weka.datagenerators
  
weka.datagenerators.classifiers.classification
  
weka.datagenerators.classifiers.regression
  
weka.datagenerators.clusterers
  
weka.estimators
  
weka.experiment
  
weka.experiment.xml
  
weka.filters
  
weka.filters.supervised.attribute
  
weka.filters.supervised.instance
  
weka.filters.unsupervised.attribute
  
weka.filters.unsupervised.instance
  
weka.filters.unsupervised.instance.subsetbyexpression
  
weka.gui
  
weka.gui.arffviewer
  
weka.gui.beans
  
weka.gui.beans.xml
  
weka.gui.boundaryvisualizer
  
weka.gui.experiment
  
weka.gui.explorer
  
weka.gui.graphvisualizer
  
weka.gui.hierarchyvisualizer
  
weka.gui.sql
  
weka.gui.sql.event
  
weka.gui.streams
  
weka.gui.treevisualizer
  
weka.gui.visualize
  
weka.gui.visualize.plugins
